# An Oxidative Stress Mechanism of Shikonin in Human Glioma Cells

**DOI:** 10.1371/journal.pone.0094180

**Published:** 2014-04-08

**Authors:** Jen-Tsung Yang, Zih-Ling Li, Jin-Yi Wu, Fung-Jou Lu, Ching-Hsein Chen

**Affiliations:** 1 Department of Neurosurgery, Chang Gung Memorial Hospital at Chiayi, Chia-Yi 613, College of Medicine, Chang Gung University, Tao-Yuan, Taiwan, ROC; 2 Department of Microbiology, Immunology and Biopharmaceuticals, College of Life Sciences, National Chiayi University, Chiayi City, Taiwan, ROC; 3 Institute of Medicine, Chung Shan Medical University, Taichung, Taiwan, ROC; Taipei Medical University, Taiwan

## Abstract

Shikonin is a quinone-containing natural product that induces the apoptotic death of some cancer cell lines in culture through increasing intracellular reactive oxygen species (ROS). Quinone-based drugs have shown potential in the clinic, making shikonin an interesting compound to study. Our previous study found that shikonin induces apoptosis in neuroblastoma by induction of ROS, but its mechanism of action and scope of activity are unknown. In this study, we investigated the mode of oxidative stress of shikonin in human glioma cells. ROS induction by shikonin was of mitochondrial origin, as demonstrated by detection of superoxide with MitoSOX Red. Pre-incubation of shikonin with inhibitors of different complexes of the respiratory chain suggested that shikonin-induced ROS production occurred via complex II. In addition, NADPH oxidase and lipooxygenase are two other main ROS-generated sites in shikonin treatment. ROS production by shikonin resulted in the inhibition of nuclear translocation of Nrf2. Stable overexpression of Nrf2 in glioma cells inhibited ROS generation by shikonin. ROS generation from mitochondrial complex II, NADPH oxidase and lipooxygenase is likely the primary mechanism by which shikonin induces apoptosis in glioma cells. These findings also have relevance to the development of certain ROS producers as anti-cancer agents. These, along with shikonin have potential as novel chemotherapeutic agents on human glioma.

## Introduction

Shikonin is a naphthoquinone compound extracted from the root of *Lithospermum erythrorhizon*. Our previous study provided evidence that shikonin causes the generation of ROS, depletion of GSH, disruption of mitochondrial transmembrane potential, upregulation of p53 and cleavage of PARP in U87MG glioma cells. Furthermore, shikonin induces downregulation of catalase and Bcl-2 as well as upregulation of SOD-1 and Bax [1]. The reactive oxygen species (ROS) generation and glutathione (GSH) depletion resulting from shikonin trigger mitochondrial transmembrane potential disruption. ROS production was partly reliant on p53 upregulation with shikonin treatment [1]. In addition, shikonin has the ability to generate large amounts of intracellular ROS during the early phase of apoptotic progression and is consequently accompanied with disturbance of mitochondrial transmembrane potential in hepatoma SK-Hep-1 cells [2]. These results suggest that a ROS-mediated oxidative stress induced by shikonin is the critical event involved in the induction of apoptosis in glioma and hepatoma cells [2].

Cell apoptosis is started by extracellular and intracellular signals through two main pathways, the death receptor- or mitochondria-mediated pathway. Numerous pathologies can result from oxidative stress-induced apoptotic signaling that results from ROS overproduction and/or antioxidant reduction, interruption of intracellular redox homeostasis, and irreversible oxidative modifications of lipid, protein or DNA [3]. The major sources of intracellular ROS include the radical-generating enzymes xanthine/xanthine oxidase [4], NADPH oxidase [5], and the phospholipase A2-activated arachidonic acid metabolism [6]. The mitochondrial respiratory chain also is a major source of intracellular ROS [7]. In the mitochondrial respiratory chain, the electrons flow through different complexes including complex I NADH reductase, complex II succinate dehydrogenase, complex III cytochrome c reductase, complex IV cytochrome c oxidase and complex V ATP synthase. Inhibition of the mitochondrial respiratory chain reduces the Δψ_m_, which facilitates the formation of the mitochondrial permeability transition pore. This permeabilization of the inner mitochondrial membrane induced by apoptotic agents is considered to be one of the mechanisms by which pro-apoptotic proteins are released from mitochondria [8]. Oxidative stress-inducing agents have been shown to increase the permeability of the inner mitochondrial membrane, and antioxidants effectively prevented mitochondrial membrane permeabilization, suggesting a relationship between ROS production, mitochondrial membrane permeabilization and apoptosis [9]–[12]. The main sources of intracellular ROS production induced by shikonin in glioma cells, and whether these sources of intracellular ROS provide a critical event that induces apoptosis are not clear so far.

In order to gain a better understanding of the oxidative stress effects on shikonin treatment in glioma cells, two human glioma cell lines: U87MG (high-grade glioma) and Hs683 (low-grade glioma) were used in this study. The aim of the present study was to compare the oxidative stress status in high-grade glioma (U87MG) and low-grade glioma (Hs683), to determine the main sources of intracellular ROS production induced by shikonin in glioma cells and to evaluate whether these sources of intracellular ROS provide a critical event to induce apoptosis in human glioma cells.

## Materials and Methods

### Cell lines and reagents

Two human glioma cell lines, U87MG and Hs683, were obtained from the Bioresource Collection and Research Center (Hsinchu, Taiwan). Dulbecco's modified Eagle's medium (DMEM) and fetal bovine serum (FBS) were obtained from Hyclone (South Logan, UT) and Biological Industries (South Logan, UT), respectively. Primary antibodies were obtained from Santa Cruz Biotechnology, Inc. (Santa Cruz, CA). Shikonin was purchased from Calbiochem (Merck KGaA, Darmstadt, Germany). The purity of shikonin was >97% as assessed by TLC. All chemicals were purchased from Sigma Chemical Co. (St. Louis, MO).

### Cell culture and treatment

U87MG and Hs683 cells were cultured in DMEM containing 10% FBS. The shikonin used in this study was purchased from Calbiochem. Shikonin was prepared by dissolving the shikonin powder in DMSO, yielding a stock solution. Different concentrations (μM) of shikonin for gliomas treatment were prepared in the cultured medium. Control cells were incubated with a volume of DMSO equal to that added to the cultures that received shikonin.

### DNA damage and cell cycle analysis

DNA damage and cell cycling were measured with PI staining and flow cytometry. After treatment, cells were collected, washed with phosphate buffered saline (PBS), fixed in PBS-methanol (1∶2, volume/volume) solution, and then maintained at 4°C for at least 18 h. After 1 wash with PBS, the cell pellets were stained with a PI solution containing PBS, PI (40 μg/mL), and DNase-free RNase A (40 μg/mL) for 30 min at room temperature in the dark. The cell pellets were then analyzed using a Becton-Dickinson FACSan flow cytometer (Franklin Lakes, NJ). Propidium iodide (PI) is an intercalating agent and a fluorescent molecule that stains double-stranded DNA. In methanol-fixed cells, the PI molecules translocate into the nucleus and bind to the double-stranded DNA. The PI fluorescent intensity in DNA-damaged cells was weaker than that of cells in the G1 phase. The percentage of DNA-damaged cells was characterized as the percentage of cells in the SubG1 region of the DNA distribution histograms. A minimum of 1×10^4^ cells was counted per sample.

### Measurement of intracellular ROS by flow cytometry

Production of various intracellular ROS was detected by flow cytometry using 10 μM 2′,7′-dichlorodihydrofluorescein-diacetate (DCFH-DA) for ROS, 10 μM hydroethidium (HE) for superoxide, 5 μM hydroxyphenyl fluorescein (HPF) for hydroxyl radical or 1 μM 4,5-diaminofluorescein-2 (DAF-2) for nitric oxide. After treatment, cells were washed once with PBS, treated with various probes for 30 min in the dark, washed again with PBS, collected by centrifugation, and then suspended in PBS. Various intracellular ROS levels were evaluated by a Becton-Dickinson FACSan flow cytometer.

### Measurement of intracellular mitochondrial superoxide production by flow cytometry

Production of mitochondrial superoxide was detected by flow cytometry using a MitoSOX Red probe. After treatment, cells were washed once with PBS, treated with 5 μM MitoSOX Red for 30 min in the dark, washed again with PBS, collected by centrifugation, and then suspended in PBS. Cells were analyzed by the flow cytometer with 488 nm excitation to measure oxidized MitoSOX Red, and a 580/22-nm barrier filter using a Becton-Dickinson FACSan flow cytometer was used for detection.

### Overexpression of Nrf2 in U87MG cells

Full-length nuclear factor-erythroid 2 p45-related factor (Nrf2) cDNA was generated by polymerase chain reaction (PCR) and subcloned into the pCMV6-XL5 expression vector (OriGene Technologies Inc., Rockville, MD). The full-length sequence was determined by automatic sequencing (ABI). The pCMV6-XL5-Nrf2 plasmid was transfected into cells using the Amaxa Cell Line Nucleofector Kit T (Lonza, VCA-1002) for 4-8 hours, and then changed fresh medium with 500 μg/ml G418 selection for two weeks. After removing the supernatant completely, cells were harvested and their nuclear proteins were extracted to determine the expression level of Nrf2 at specific time intervals by means of western blotting.

### Preparation of nuclear protein extracts from U87MG cells for Nrf2 analysis

After treatment, glioma cells were washed with cold PBS and suspended in 0.1 mL of hypotonic lysis buffer containing protease inhibitors for 30 min. Cells were then lysed with 3.2 μl of 10% Nonidet P-40. The homogenate was centrifuged, and the supernatant containing the cytoplasmic extracts was stored frozen at −80 °C. The nuclear pellet was resuspended in 25 μl of ice-cold nuclear extraction buffer. After 30 min of intermittent mixing, the extract was centrifuged and the supernatants containing the nuclear extracts were secured. Nuclear Nrf2 expression was evaluated by western blotting.

### Western blotting analysis

After treatment, cells were washed with PBS, resuspended in a protein extraction buffer for 10 min, and centrifuged at 12,000 g for 10 min at 4°C in order to obtain total extracted proteins (supernatant). Protein concentrations were measured using a protein assay reagent (Bio-Rad, Richmond, CA). The extracted cellular proteins were boiled in loading buffer, and an aliquot corresponding to 50–100 μg of protein was separated on a 12% SDS-polyacrylamide gel. After electrophoresis, proteins were electrotransferred onto a polyvinylidene fluoride transfer membrane. After blotting, the membranes were incubated with various primary antibodies overnight and then washed with PBST solution (0.05% Tween 20 in PBS). Following washing, the secondary antibodies labeled with horseradish peroxidase were added to the membrane for 1 h; they were then washed with PBST solution (0.05% Tween 20 in PBS). The antigen-antibody complexes were detected by enhanced chemiluminescence (Amersham Pharmacia Biotech, Piscataway, NJ) using a chemiluminescence analyzer.

### Detection of cytosolic cytochrome c

After treatment, cells were washed with PBS, resuspended in homogenization buffer (0.25M sucrose, 10 mM HEPES and 1 mM ethylene glycol bis(b-aminoethylether)-N,N,N′,N′-tetraacetic acid, pH 7.4) and subjected to 50 strokes of homogenization in a glass homogenizer. The homogenates were centrifuged at 1000 g for 15 min at 4 °C to precipitate nuclei and unbroken cells. The supernatant was then centrifuged at 10,000 g for 15 min at 4 °C to obtain cytosolic fraction (supernatant). The cytochrome c in cytosolic fraction was evaluated by Western blotting analysis.

### Detection of mitochondrial membrane potential

After treatment, cells were collected by trypsinization and stained with 5 μM rhodamine 123 for 30 min; they were then washed once with PBS. The mitochondrial membrane potential (indicated by the fluorescence level of rhodamine 123) was analyzed with a Becton-Dickinson FACS-Calibur flow cytometer with the excitation and emission wavelengths set at 488 and 520 nm, respectively.

### Detection of caspase 3, 8 and 9 activities by flow cytometry

Caspase 3, 8 and 9 activities were detected by an Apo-one™ homogeneous caspase-3 assay kit. The caspase substrates, Z-DEVD-R110 for caspase 3, FITC-IETD-FMK for caspase 8, and FITC-LEHDFMK for caspase 9, were diluted with a buffer to make the desired concentrations of various homogeneous substrate reagents. After drugs treatment, the cells were washed once with PBS, detached by trypsinization, and collected by centrifugation. Aliquot 1×106 cells were suspended in a DMEM medium, and then various homogeneous substrate reagents were added to the cells, maintaining a 1∶1 ratio of reagent to cell solution. After 1 h of incubation at 37°C, the cells were washed once with PBS, collected by centrifugation, and suspended in PBS. Substrate cleavage to release free R110 or FITC fluorescence intensity was detected in a Becton–Dickinson FACS-Calibur flow cytometer with excitation wavelength set at 488 nm and emission wavelength at 520 nm.

### HPLC quantitative analysis of shikonin in cells

For HPLC analysis using with RP-C_18_ column (4.6 mm×250 mm, 5 μm, Merck, Germany), methanol–water (85∶15, v/v) was used as the mobile phase. The mobile phase was filtered through a 0.47 μm membrane filter and then deaerated ultrasonically prior to use. Shikonin was quantified by a UV detector at the wavelength of 220 nm following HPLC separation. Flow rate was 1.0 mL/min, the injection volume was 10 μl and the column temperature was maintained at 25 °C. The chromatographic peak of the analyte was confirmed by comparing its retention time with the reference standard. Quantification was carried out by the integration on area under curve (AUC) of the peak using external standard method. The working calibration curve based on shikonin standard solutions showed good linearity over the range of 2–10 μM. The regression line for shikonin was y = 0.20077x – 0.13267 (R^2^ = 0.988), where y is the peak area of shikonin, and x is the concentration (μM).

### Statistical analysis

Data are presented as the mean (SD) of at least 3 independent experiments and were analyzed using Student's *t*-test. A *P* value <0.05 was considered statistically significant.

## Results

### Multiple sources of ROS induced by shikonin in glioma cells

**Figure 1:**
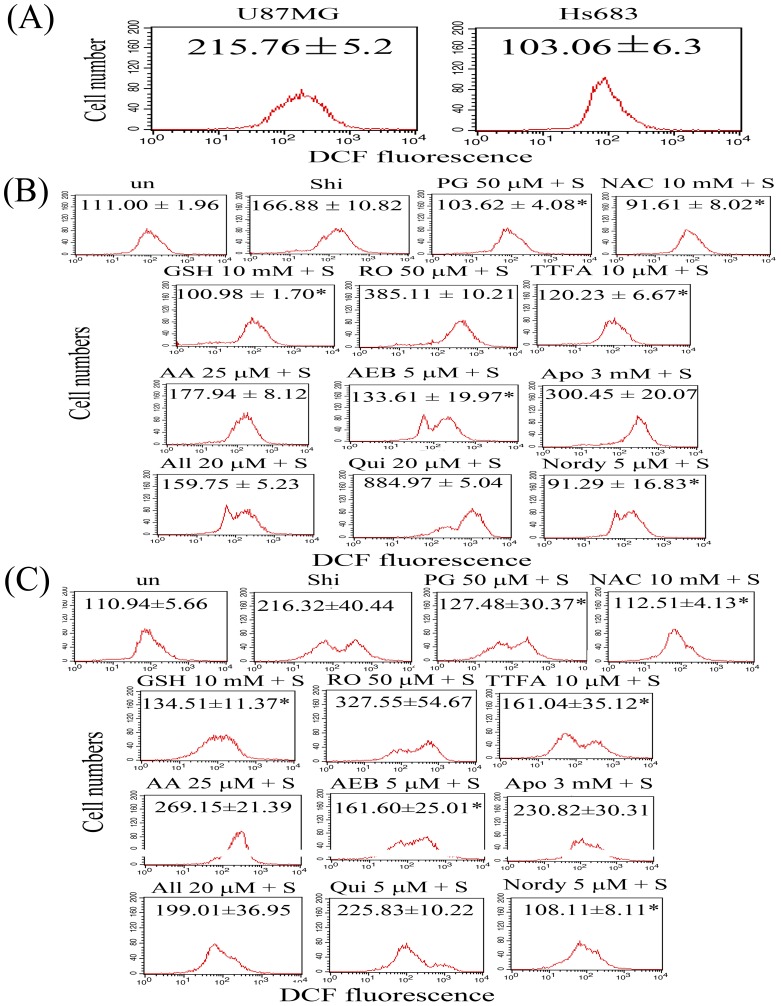
Evaluation of intracellular ROS in shikonin-treated Hs683 cells and U87MG cells. Cells were plated in 60-mm culture dishes. The culture medium was replaced with fresh medium when the cells reached 80% confluence. (A) Intracellular ROS of untreated Hs683 and untreated U87MG cells was detected by flow cytometry using DCFH-DA staining. (B) Hs683 cells and (C) U87MG cells were treated with 8 μM shikonin (Shi) alone for 2 h or pretreated with 50 μM rotenone (RO, a complex I inhibitor), 10 μM 2-ethenoyltrifluoroacetone (TTFA, a complex II inhibitor), 25 μM antimycin A (AA, a complex III inhibitor), 3 μM apocynin (Apo, a NADPH oxidase inhibitor), 5 μM 4-(2-Aminoethyl)benzenesulfonyl fluoride hydrochloride (AEB, a NADPH oxidase inhibitor), 10 μM allopurinol (All, a xanthine oxidase inhibitor), 10 μM quinacrine (Qui, phospholipase A2 inhibitors) or 5 μM Nordydihydroguaiaretic acid (Nordy, lipoxygenase inhibitor), 10 mM *N*-Acetylcysteine (NAC), 10 mM glutathione (GSH), and 50 μM propyl gallate (PG) for 1 h, followed by 8 μM shikonin (S) treatment for 2 h. Production of intracellular ROS was detected by flow cytometry using DCFH-DA staining. The intracellular fluorescence of dichlorofluorescein (DCF) was measured using a Becton-Dickinson FACScan flow cytometer. Data in each panel represent the DCF fluorescence intensity within cells. The values shown are mean (SD) (n = 5–8 samples per experiment). Significant differences from the shikonin group show *P*<0.05 (*).

U87MG cells are a grade IV glioma cells and Hs683 cells are a grade I∼II cells. We first demonstrated that the intracellular ROS level in the untreated U87MG cells is larger than the untreated Hs683 cells (Figure 1A). It seems to explain that U87MG cells have a high ROS generated systems. Once shikonin treatment, the ROS generation in U87MG cells may be larger than in Hs683 cells. To evaluate the ROS sources of shikonin treatment in U87MG and Hs683 glioma cells, cells were pre-incubated with 50 μM rotenone (RO, a complex I inhibitor), 10 μM 2-ethenoyltrifluoroacetone (TTFA, a complex II inhibitor), 25 μM antimycin A (AA, a complex III inhibitor), 3 μM apocynin (Apo, a NADPH oxidase inhibitor), 5 μM 4-(2-Aminoethyl)benzenesulfonyl fluoride hydrochloride (AEB, a NADPH oxidase inhibitor), 10 μM allopurinol (All, a xanthine oxidase inhibitor), 10 μM quinacrine (Qui, phospholipase A2 inhibitors) or 5 μM Nordydihydroguaiaretic acid (Nordy, lipoxygenase inhibitor), or various antioxidants such as 10 mM *N*-Acetylcysteine (NAC), 10 mM glutathione (GSH), and 50 μM propyl gallate (PG), followed by co-incubation with shikonin. After treatment, the intracellular ROS production was measured using the DCFH-DA probe. A significant increase in intracellular ROS after shikonin treatment was observed in both cell lines (Figure 1). Shikonin-induced intracellular ROS production, measured using the DCFH-DA probe and flow cytometry, was significantly scavenged when cells were pre-incubated for 1 h with PG, NAC, GSH, TTFA, AEB, and Nordy in Hs683 and U87MG cells (Figure 1). However, RO, AA, Apo, All and Qui did not scavenge the shikonin-induced intracellular ROS production. These results indicated that there were 3 main sources of ROS, including mitochondrial complex II, NADPH oxidase, and lipoxygenase, induced by shikonin treatment in glioma cells.

### Simultaneous detection of mitochondrial superoxide production in shikonin-treated human glioma cells

**Figure 2:**
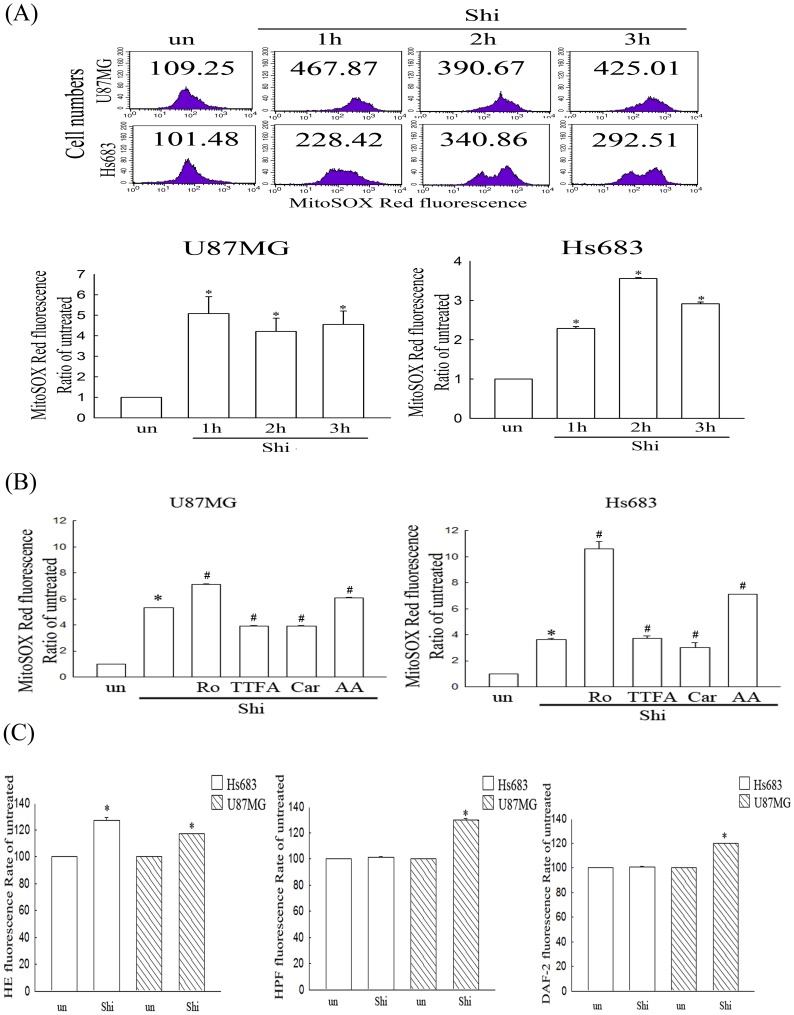
Evaluation of mitochondrial superoxide and various ROS species in shikonin-treated U87MG cells and Hs683 cells. Cells were plated in 60-mm culture dishes. The culture medium was replaced with fresh medium when the cells reached 80% confluence. (A) Cells were treated with 4 μM shikonin alone for 1, 2 and 3 h; (B) Cells were treated with 4 μM shikonin alone for 2 h or pretreated with 50 μM rotenone (RO, a complex I inhibitor), 10 μM 2-ethenoyltrifluoroacetone (TTFA, a complex II inhibitor), 10 μM carboxin (Car, a complex II inhibitor), and 25 μM antimycin A (AA, a complex III inhibitor) for 1 h, followed by 4 μM shikonin treatment for 2 h. Production of mitochondrial superoxide was detected by flow cytometry using Mitosox Red staining. The intracellular fluorescence of Mitosox Red was measured using a Becton-Dickinson FACScan flow cytometer. (C) Hs683 and U87MG glioma cells were treated with 8 μM shikonin (Shi) for 2 h. Production of superoxide, OH radical and nitric oxide were detected by flow cytometry using hydroethidium (HE), hydroxyphenyl fluorescein (HPF) or 4,5-diaminofluorescein-2 (DAF-2) staining, respectively. Data in each panel represent the Mitosox Red fluorescence intensity within cells. The values shown are mean (SD) (n = 5–8 samples per experiment). Significant differences from the untreated group and shikonin-treated group show *P*<0.05 (*) and *P*<0.05 (#), respectively.

A novel fluoroprobe, MitoSOX Red, was introduced for selective detection of superoxide in the mitochondria of live cells and was validated for flow cytometry. We further measured acute mitochondrial superoxide formation by flow cytometry in shikonin-treated glioma cells using MitoSOX Red staining. As shown in Figure 2A, shikonin rapidly increased mitochondrial superoxide generation at 1, 2 and 3 h in U87MG and Hs683 glioma cells measured by flow cytometry, indicating mitochondria provides a ROS source in shikonin treatment of glioma cells. Moreover, both mitochondrial complex II inhibitors, TTFA and carboxin, partially inhibited the MitoSOX Red fluorescence induced by shikonin in U87MG cells (Figure 2B), further confirming that mitochondrial complex II is the ROS-generated site in shikonin treatment.

### The species of ROS induced by shikonin

To clarify the species of ROS induced by shikonin, we used hydroethidium (HE), hydroxyphenyl fluorescein (HPF) and 4,5-diaminofluorescein-2 (DAF-2) to detect superoxide, OH radical and nitric oxide, respectively, in shikonin treated glioma cells. Figure 2C shows that the fluorescence of HE, HPF and DAF-2 increased in shikonin-treated U87MG glioma cells. However, only HE fluorescence was increased in shikonin-treated Hs683 glioma cells. It is indicating that superoxide, OH radical and nitric oxide are the three main ROS species in U87MG cells and superoxide is the main ROS species in Hs683 cells induced by shikonin treatment.

### Shikonin inhibits nuclear Nrf2 expression in glioma cells

**Figure 3:**
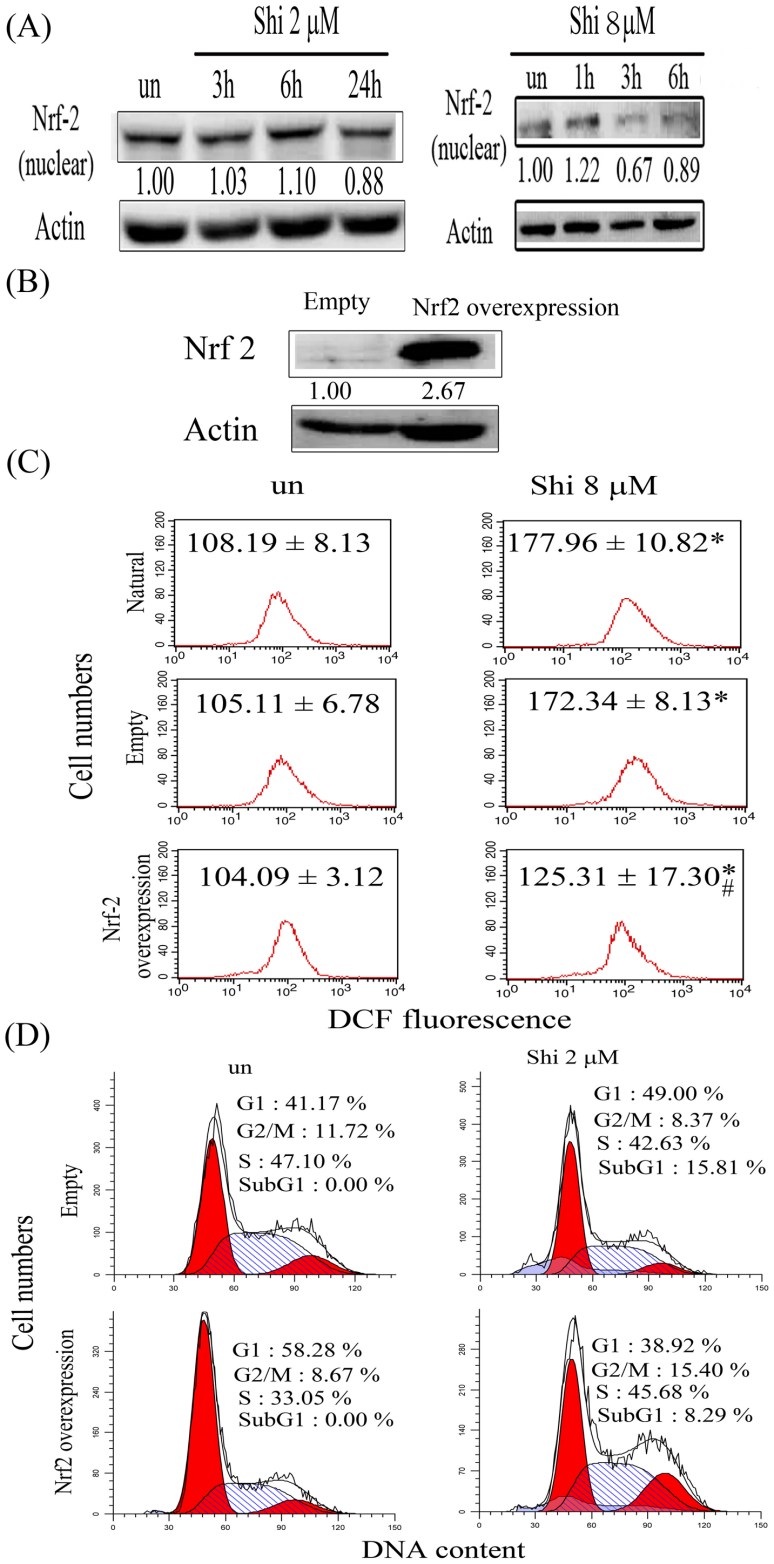
The expression of nuclear Nrf2 in shikonin-treated U87MG cells. (A) Cells were plated in 60-mm culture dishes at 80% confluence and then treated with 2 μM or 8 μM shikonin for the indicated time points. After treatment, nuclear protein was extracted to assess Nrf2 expressions. Fifty micrograms of protein was loaded onto a 12% SDS-polyacrylamide gel and evaluated by western blotting. These experiments were performed at least 3 times; a representative experiment is presented. (B) U87MG cells were transfected with pCMV6-XL5 empty plasmid or pCMV6-XL5-nuclear factor-Nrf2 plasmid, and the stable overexpression of nuclear Nrf2 was assessed by western blotting. (C) Natural, empty plasmid-transfected and Nrf2 plasmid-transfected U87MG cells were treated with 8 μM shikonin for 3 h. Production of intracellular ROS was detected by flow cytometry using DCFH-DA staining. The intracellular fluorescence of dichlorofluorescein (DCF) was measured using a Becton-Dickinson FACScan flow cytometer. Data in each panel represent the DCF fluorescence intensity within cells. The values shown are mean (SD) (n = 5–8 samples per experiment). Significant differences from the untreated group show *P*<0.05 (*) and empty shikonin-treated group show *P*<0.05 (#). (D) Empty plasmid-transfected (Empty) and Nrf2 plasmid-transfected (Nrf2 overexpression) U87MG cells were treated with 2 μM shikonin for 24 h. All samples were subsequently processed for cell cycle analysis. These experiments were performed at least 3 times; a representative experiment is presented.

Nrf2 is a redox signaling transcription factor; it regulates intracellular oxidative stress during redox status imbalance. Nrf2 also transcripts many phase II enzymes to against oxidative stress during treatment with anticancer agents. Because shikonin induced early intracellular ROS overproduction in glioma cells, the nuclear Nrf2 expression during shikonin treatment of glioma cells was evaluated by western blotting. As shown in Figure 3A, the nuclear Nrf2 expression was slightly increased at 6 h and inhibited after 24 h by shikonin treatment in U87MG cells, suggesting shikonin-induced ROS overproduction damages the defensive Nrf2 system at a later stage and might lead to subsequent cell death. We further used higher concentration of shikonin (8 μM) to treat glioma cells for 1, 3 and 6 h and detected the Nrf2 translocation. As shown in Figure 3A, the Nrf2 translocation is inhibited by shikonin treatment in glioma cells, indicating that shikonin interfere Nrf2 translocation to the nuclear. To further evaluate the relationship between ROS overproduction and nuclear Nrf2 inhibition during shikonin treatment, U87MG cells were stable overexpressed nuclear Nrf2 by transfecting with pCMV6-XL5-Nrf2 plasmid and the intracellular ROS on shikonin treatment was detection. Figure 3B shows that nuclear Nrf2 expression increased 2.67-fold in U87MG cells after transfection with pCMV6-XL5-Nrf2. Nrf2 overexpression partially scavenged the intracellular ROS and inhibited the apoptosis on shikonin treatment (Figures 3C and 3D), which confirmed that the early intracellular ROS during shikonin treatment participated in the damage to the antioxidant defensive system in glioma cells.

### The expression of phase II enzymes on shikonin treatment in glioma cells

**Figure 4:**
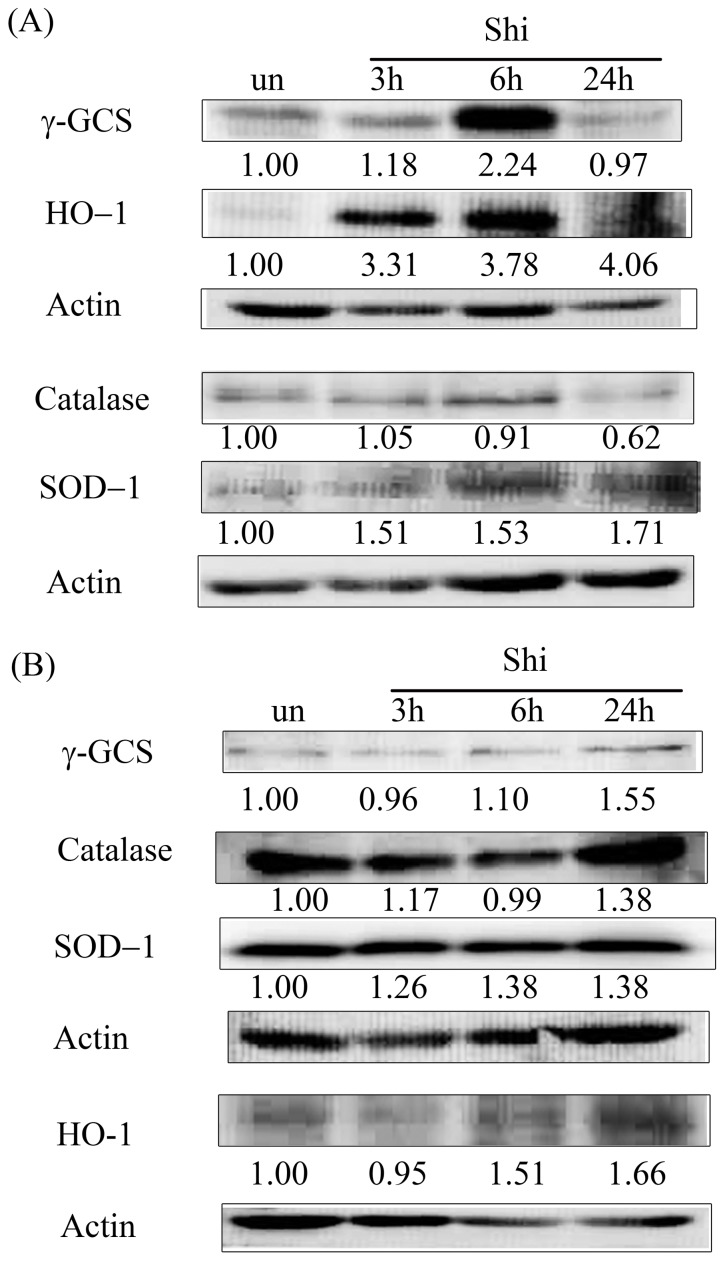
The expressions of γ-GCS, HO-1, catalase and SOD-1 in shikonin-treated (A) U87MG and (B) Hs683 cells. Cells were plated in 60-mm culture dishes at 80% confluence and then treated with 2 μM shikonin for 3, 6, or 24 h. After treatment, total protein was extracted to assess various protein expressions. Fifty micrograms of protein were loaded onto a 12% SDS-polyacrylamide gel and evaluated by western blotting. These experiments were performed at least 3 times; a representative experiment is presented.

Nrf2 regulates the expression of phase II enzymes. We further evaluated the expression of phase II enzymes with shikonin treatment in glioma cells. In U87MG cells, the expressions of γ-GCS were increased at 3 and 6 h and decreased at 24 h of shikonin treatment (Figure 4A). The expressions of HO-1 and SOD-1 were increased at all treated time points. The expression of catalase was inhibited by shikonin at 6 and 24 h. The increased expression of HO-1, γ-GCS and SOD-1 seemed to be accompanied with the Nrf2 increase at the early stage of shikonin treatment. In Hs683 cells, the expressions of γ-GCS and HO-1 were slightly inhibited by shikonin at 3 h (Figure 4B). However, shikonin increased the expressions of γ-GCS, catalase, SOD-1 and HO-1 at 24 h.

### The sources of ROS induced by shikonin provoke apoptosis in glioma cells

**Figure 5:**
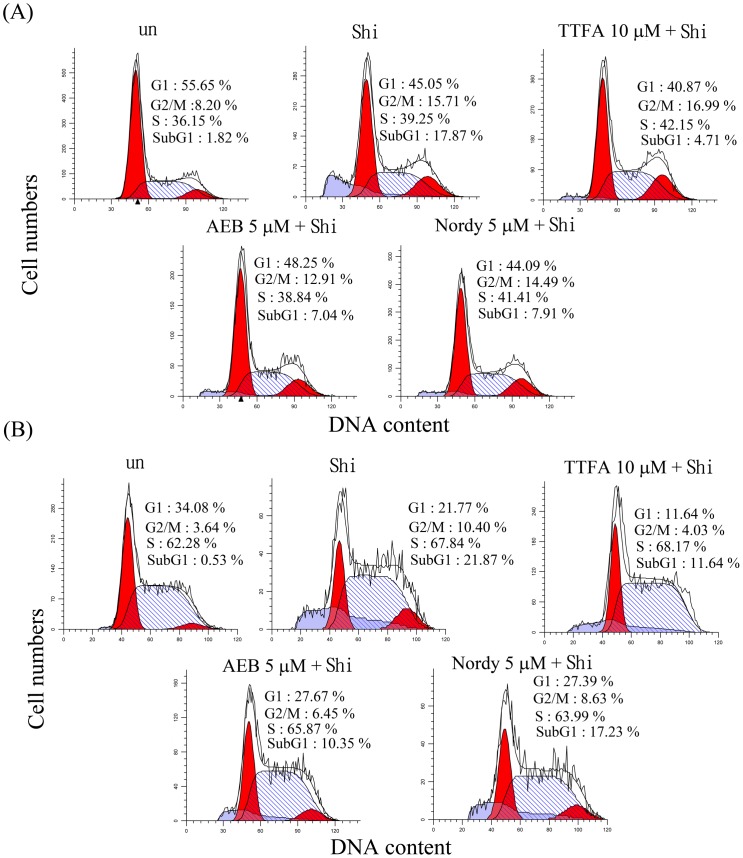
Evaluation of the critical ROS events in shikonin-induced apoptosis. (A) U87MG and (B) Hs683 cells were treated with 2 μM shikonin (Shi) alone for 24 h or pretreated with 10 μM 2-ethenoyltrifluoroacetone (TTFA, a complex II inhibitor), 5 μM 4-(2-Aminoethyl)benzenesulfonyl fluoride hydrochloride (AEB, a NADPH oxidase inhibitor), and 5 μM Nordydihydroguaiaretic acid (Nordy, lipoxygenase inhibitor for 1 h, followed by 2 μM shikonin treatment for 24 h. All samples were subsequently processed for cell cycle analysis. These experiments were performed at least 3 times; a representative experiment is presented.

Our previous study demonstrated that shikonin could induce apoptosis in glioma cells. To evaluate whether the ROS production from mitochondrial complex II, NOX, or lipoxygenase induced by shikonin treatment could provoke apoptosis, U87MG and Hs683 glioma cells were pre-incubated with 10 μM TTFA, 5 μM AEB and 5 μM Nordy for 1 h, followed by co-incubation with shikonin for 24 h. After treatment, apoptosis (SubG1) was measured using PI staining and flow cytometry. A significant increase of SubG1 percentages after shikonin treatment was observed in U87MG and Hs683 glioma cells (Figure 5). Three inhibitors of ROS-produced enzymes could partially inhibit the SubG1 percentages in shikonin treatment, suggesting the ROS produced from mitochondrial complex II, NOX, and lipoxygenase could result in apoptosis in glioma cells.

### Shikonin can disrupt the mitochondria function and can release cytochrome c to the cytosol

**Figure 6:**
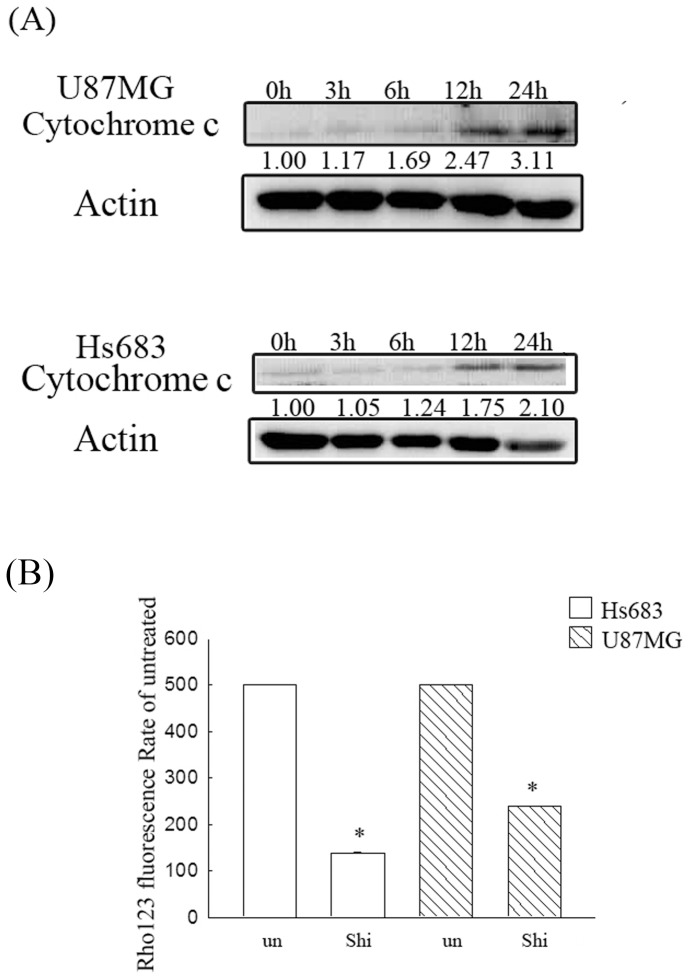
Evaluation of the cytosolic cytochrome c and mitochondrial membrane potential in shikonin treatment. (A) U87MG and Hs683 cells were treated with 2 μM shikonin for 3, 6, 12 or 24 h. After treatment, cytosolic protein was extracted to assess cytochrome c expression. Fifty micrograms of protein were loaded onto a 12% SDS-polyacrylamide gel and evaluated by western blotting. These experiments were performed at least 3 times; a representative experiment is presented. (B) U87MG and Hs683 cells were treated with 2 μM shikonin for 6 h. After treatment, cells were stained with 5 μM rhodamine 123 (Rho 123) for 30 min. The rhodamine 123 fluorescence represented the mitochondrial membrane potential was detected by flow cytometry. Significant differences from the untreated group show *P*<0.05 (*).

To clarify whether shikonin could directly disrupt the mitochondria function and could cytochrome c release to the cytosol, the mitochondrial membrane potential were detected by rhodamine 123 staining and flow cytometry and the cytosolic proteins after shikonin treatment were extracted and detected the cytochrome c release by western blotting. As shown in Figure 6, the cytochrome c release is increased at 12 and 24 h of shikonin treatment (Figure 6A) and the mitochondrial membrane potential was decreased after 6 h of shikonin treatment (Figure 6B), suggesting that shikonin could directly disrupt the mitochondria function and could cytochrome c release to the cytosol.

### Shikonin induces intrinsic and extrinsic apoptosis mechanism

**Figure 7:**
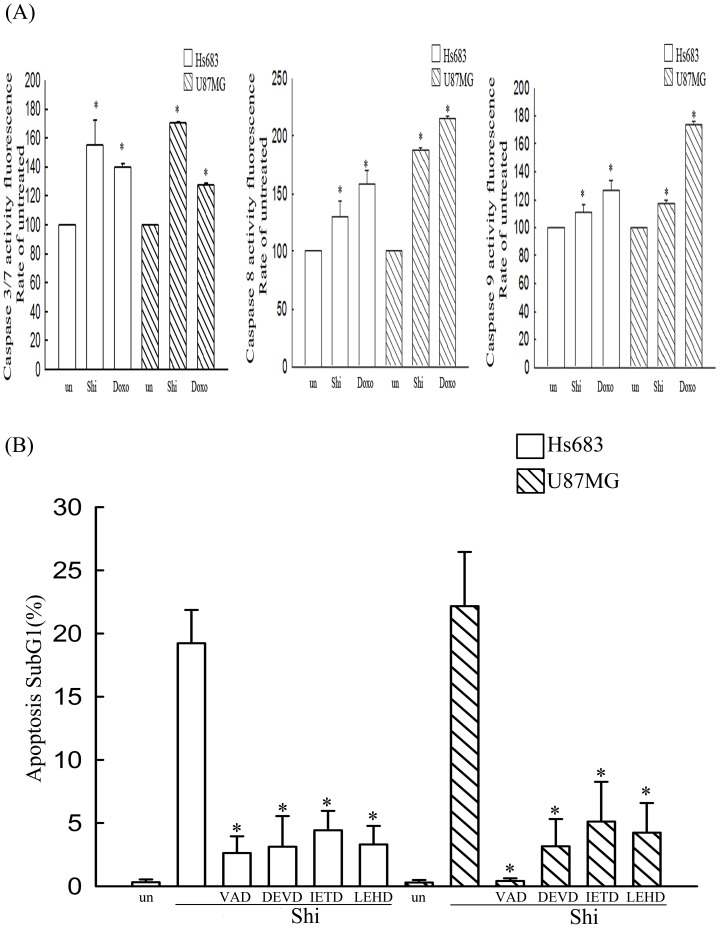
Evaluation of the caspases activity in shikonin treatment. (A) Hs683 and U87MG cells were treated with 2 μM of shikonin (shi) or an apoptosis positive agent, doxorubicin (doxo) for 6 h. After drugs treatment, the cells were washed once with PBS, detached by trypsinization, and collected by centrifugation. Aliquot 1×10^6^ cells were suspended in a DMEM medium, and then various homogeneous substrate reagents, Z-DEVD-R110 for caspase 3/7, FITC-IETD-FMK for caspase 8, and FITC-LEHD-FMK for caspase 9, were added to the cells, maintaining a 1∶1 ratio of reagent to cell solution. After 1 h of incubation at 37 °C, the cells were washed once with PBS, collected by centrifugation, and suspended in PBS. The substrate cleavage to release free R110 or FITC fluorescence intensity in cells was analyzed using a Becton–Dickinson FACS-Calibur flow cytometer. The values shown are mean (SD) (n = 5–8 of individual experiments). Significant differences from the untreated group (un) are P<0.05 (*). (B) Hs683 and U87MG cells were treated with 2 μM shikonin (Shi) alone for 24 h or pretreated with 100 μM of Z-VAD-FMK (a cell-permeant pan caspase inhibitor), Z-DEVD-FMK (a cell-permeable, irreversible caspase-3 inhibitor), Z-IETD-FMK (a cell-permeable, irreversible caspase-8 inhibitor) or Z-LEHD-FMK (a cell-permeable, irreversible caspase-9 inhibitor) for 1 h, followed by 2 μM shikonin treatment for 24 h. All samples were subsequently processed for cell cycle analysis to evaluate the percentage of apoptosis subG1 phase. The value shown are mean (SD) (n = 5–8 of individual experiments). Significant differences from the shikonin alone groups are P<0.05 (*).

We performed the caspases activity assay in shikonin treatment by flow cytometry. Shikoinin could increase caspase 3, caspase 8 and caspase 9 activities in glioma cells (Figure 7A). Moreover, we further used specific inhibitors to verify the molecular mechanism of apoptosis induced by shikonin. Our results show that the pan-caspase inhibitor and other caspase inhibitors including caspase 3 inhibitor, caspase 8 inhibitor or caspase 9 inhibitor could inhibit the apoptosis induced by shikonin (Figure 7B), indicating the molecular mechanism of apoptosis induced by shikonin is through intrinsic and extrinsic apoptosis mechanism.

### Shikonin can penetrate into cytosol of glioma cells

**Figure 8:**
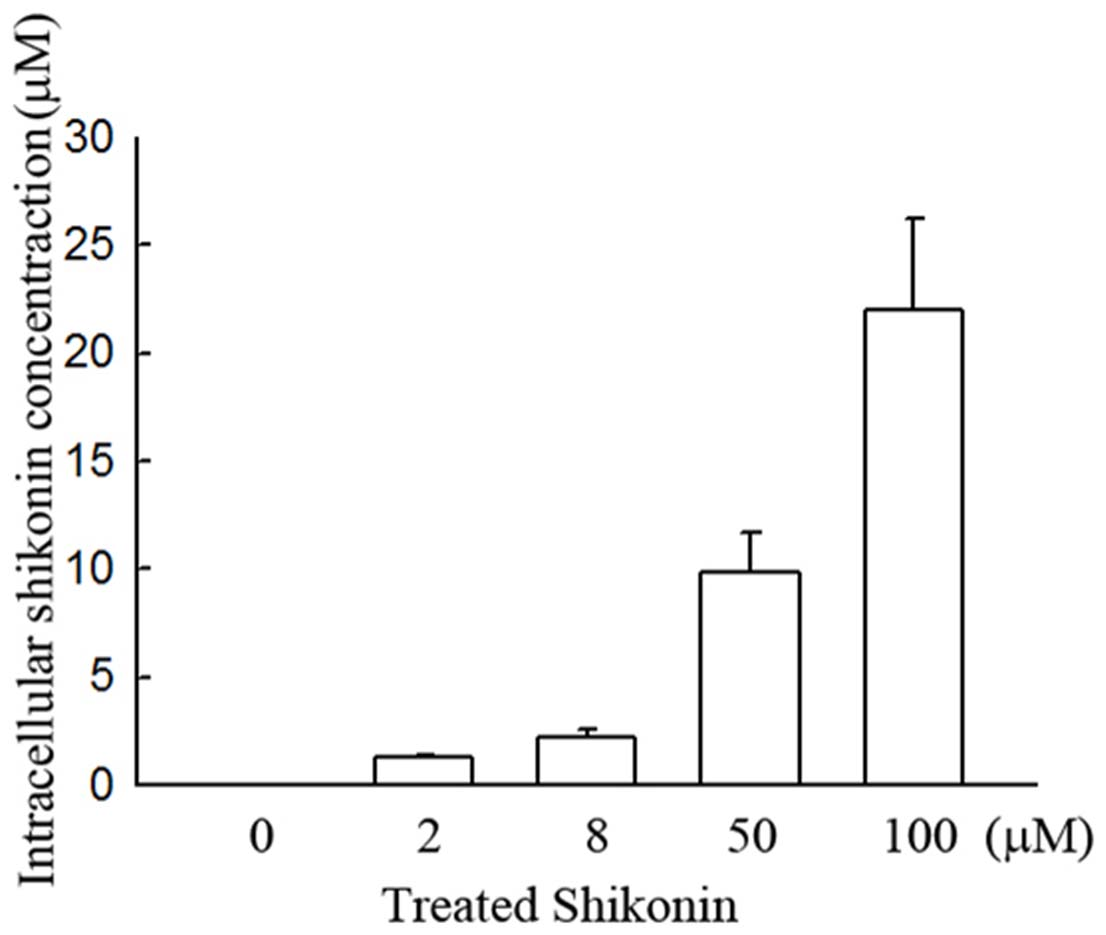
Evaluation of the intracellular shikonin concentration in shikonin-treated glioma cells. U87MG cells were treated with 2, 8, 50 and 100 μM of shikonin for 2 h. After treatment, cells were shattered by sonication and then centrifuged at 12,000 g for 10 min at 4°C. The cytosolic substances (supernatant) were evaluated the shikonin concentration detected by HPLC.

To demonstrate whether shikonin could penetrate into cytosol, the cytosolic substances after 2, 8, 50 and 100 μM of shikonin treatment for 2 h were extracted and detected the shikonin concentrations by HPLC. As shown in Figure 8, the shikonin exists in the cytosolic substances after shikonin treatment, suggesting that shikonin can penetrate into cytosol.

## Discussion

Many anti-cancer agents during the past decade have shown well established mechanisms of action that involve free radical intermediates or free radical generation. Among these anti-cancer agents, anthracyclins, most alkylating agents and platinum coordination complexes partly exert their anti-tumor activity or toxic side effects by generating oxidative stress. Drug-induced oxidative stress is enforced through enzymatic pathways related to NADPH moxidase, xanthine oxidase, lipooxygenase or the mitochondrial electron transport chain [13]. In cancer cells, the drug-induced intrinsic oxidative stress produces potent ROS-mediated cytotoxic procedures that preferentially kill tumor cells or inhibit their proliferation.

Compounds including the 1,4-naphthoquinone moiety appear ubiquitously in nature. Because of their high cytotoxicity, naphthoquinone derivatives have been studied as model compounds for the development of anti-cancer drugs performing on target cells both by the production of reactive metabolites and by directly interfering with cellular enzymes fundamental for cell proliferation [14]. For example, menadione containing a fat-soluble 2-methyl-1,4-naphtoquinones structure also has been reported to express a broad range of anticancer activity in human cells [15]–[17]. The cytotoxicity of quinone family compounds has been shown to be arbitrated by various mechanisms, including interference of redox cycling and free radical formation [18]. In all aerobic cells, ROS are derived from the metabolism of molecular oxygen and normally exist in balance with biochemical antioxidants. When this key balance is disrupted by the presence of excess ROS, antioxidant depletion or both, oxidative stress will appear [19], [20]. A growing body of data showing that chemotherapeutic agents may be displayed selectively tumor cells cytotoxicity since they enhance oxidant stress exceed their limit in these originally stressed cells [21], [22]. More and more evidence demonstrates that cytotoxic ROS signaling provokes the intrinsic mitochondrial apoptotic pathway, as indicated by exchanges in Bax/Bcl-2 ratios, which induces mitochondrial membrane potential disruption, cytochrome c release and caspase-9 activation [23], [24]. Our current results demonstrate for the first time that the mitochondrial ROS source of shikonin treatment is complex II of the electron transport chain. The shikonin-induced mitochondrial ROS might immediately destroy the mitochondrial function and then induce the intrinsic apoptosis pathway. This speculation can be confirmed by our results, that complex II inhibitor TTFA could recover the apoptosis induced by shikonin.

Succinate:quinone reductase of complex II captured a sole central point in the mitochondrial respiratory chain as a major source of electrons driving reactive oxygen species (ROS) generation [25]. For this reason, succinate:quinone reductase of complex II is an ideal pharmaceutical target for modulating ROS levels to increase ROS in cancer cells, thereby inducing cell death. Our results show that complex II, but not complex I and III, provided the ROS source in shikonin treatment of human glioma cells. This seems to be in agreement with MitoQ inducing ROS production. MitoQ, containing a quinone moiety, is a poor substrate for complexes I and III, whereas it is in fact an effective redox complex II. Additional evidence indicates that the MitoQ quinone moiety can contact the ubiquinone-binding site of complex II, but not complex I [26]. Thus, it is more likely that MitoQ was binding to the Q sites of complex II not complex I, and facilitating ROS generation from there. Our results indicated that shikonin could act as a pro-oxidant and was likely binding to complex II because TTFA prevented the shikonin-induced ROS increase. Inhibiting complex I with rotenone and complex III with antimycin A did not alter the effect of shikonin in ROS production, thus ruling out the ROS-induced effects of shikonin on complex I and complex III. These results provide real support for the binding of shikonin to complex II, where it can act as a pro-oxidant, and are consistent with ROS being produced directly and intrinsically from succinate:quinone reductase.

Our results demonstrated that shikonin-induced ROS production also came from NADPH oxidase in human glioma cells. NADPH oxidase exists in a wide range of tissues and plays a role in cellular signaling by generating ROS [27]. Another study indicated some specific quinine derivatives could modulate the activity of NADPH oxidase [28]. Due to the complex mechanisms involved in the activation of NADPH oxidases, these enzymes can be targeted at many different levels of activity [29]. Once NADPH oxidase can regulate the activities of several proteins and downstream signaling pathways, and provides the major non-mitochondrial source of ROS inside cells, it would be an important therapeutic target in cancer [28]. Our results demonstrated that inhibition of NADPH oxidase by AEB partially abolished the apoptosis effect of shikonin in glioma cells. This seems to confirm that NADPH oxidase provides the major non-mitochondrial source of ROS to induce apoptosis in glioma cells.

Shikonin has both quinone and semiquinone moieties in its molecule. In the presence of cytochrome c, superoxide anion radical formed by the rapid reaction of the semiquinone moiety of shikonin with O_2_ is responsible for the reduction reaction in mitochondria. Simultaneously, the semiquinone moiety of shikonin is altered to semiquinone radicals. The quinone moiety of shikonin can produce superoxide responsible through reactions between dioxygen and semiquinone radicals. Taken together, our data suggest that shikonin can induce the apoptosis of glioma cells, and that increased ROS generation appears to be primarily responsible for this increased cell death. Shikonin-induced ROS production in glioma cells mainly takes place in the mitochondria, more precisely, at complex II of the mitochondrial respiratory chain. Other major non-mitochondrial sources of ROS inside cells are NADPH oxidase and lipooxygenase. This study may provide new insight for the investigation of oxidative stress mechanisms and the apoptosis-related ROS pathway in shikonin treatment in human glioma cells.
